# Supplementary material for: Virus Detection by CRISPR-Cas9-Mediated Strand Displacement in a Lateral Flow Assay
Source: ACS Appl Bio Mater. 2025 Apr 24;8(5):4221–9. doi: 10.1021/acsabm.5c00307 (PMC12093361; doi:10.1021/acsabm.5c00307)
Supplement: Supplementary file 1 — mt5c00307_si_001.pdf [file mt5c00307_si_001.pdf]

Supporting Information

Virus detection by CRISPR-Cas9-mediated strand displacement in a lateral flow assay

Roser Montagud-Martínez[1,†], Rosa Márquez-Costa[1,†], Raúl Ruiz[1], Adrià Martínez-Aviñó[2], Rafael Ballesteros-Garrido[3], David Navarro[4,5], Pilar Campins-Falcó[2], and Guillermo Rodrigo[1,*]

1. Institute for Integrative Systems Biology (I2SysBio), CSIC – University of Valencia, 46980 Paterna, Spain
2. Department of Analytical Chemistry, School of Chemistry, University of Valencia, 46100 Burjassot, Spain
3. Department of Organic Chemistry, School of Pharmacy, University of Valencia, 46100 Burjassot, Spain
4. Microbiology Service, Clinic University Hospital, INCLIVA Biomedical Research Institute, 46010 Valencia, Spain
5. Department of Microbiology, School of Medicine, University of Valencia, 46010 Valencia, Spain
† Equal contribution
* Correspondence: guillermo.rodrigo@csic.es

A

B

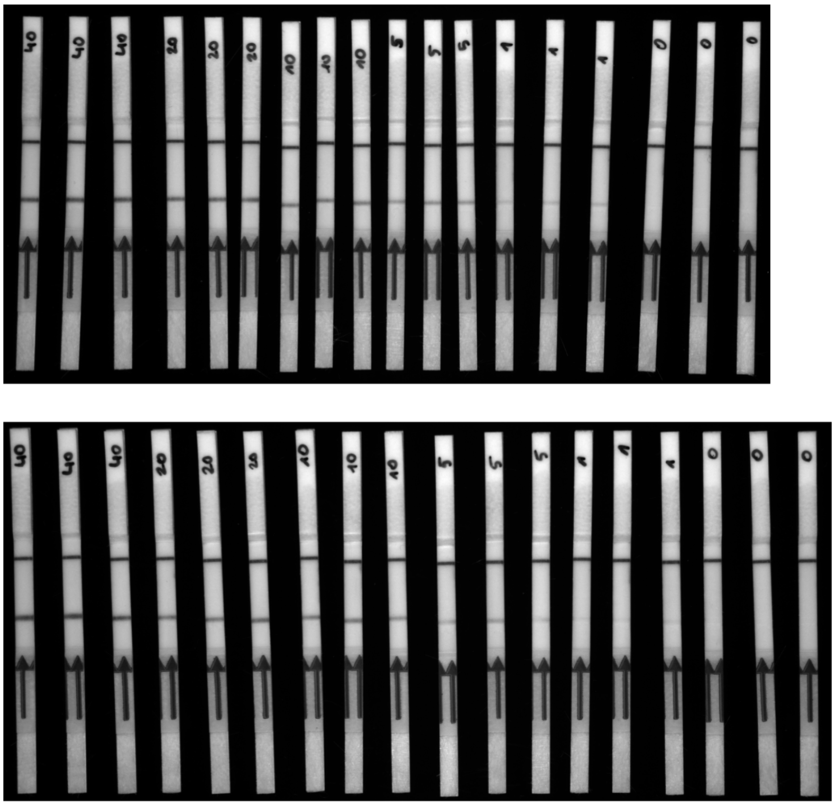

Fig. S1: Image of replicate LFA strips in the detection of the dsDNA amplicons from the N and E genes of SARS-CoV-2. From right to left, increasing concentrations of dsDNA (from 0 to 40 nM). A) N gene amplicon. B) E gene amplicon.

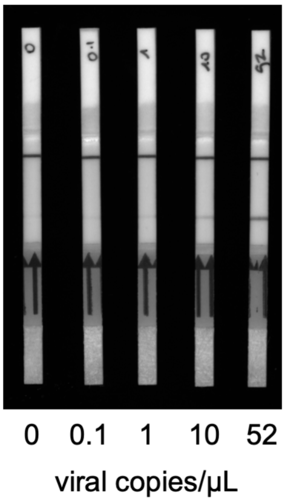

Fig. S2: Image of representative LFA strips to assess the limit of detection of the method, targeting the E gene of SARS-CoV-2 (RNA genome). Final concentrations in the RT-RPA reaction.

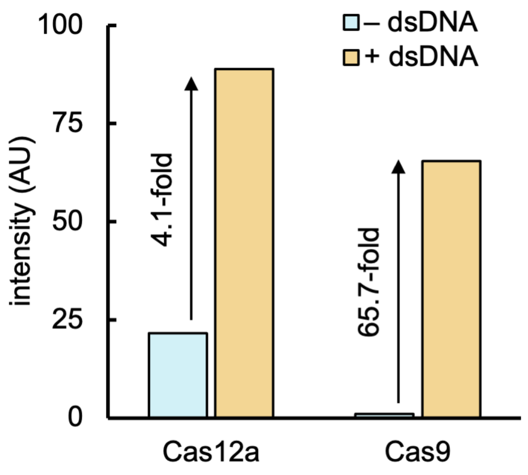

Fig. S3: Quantified intensity of the test line band with and without dsDNA input (N gene amplicon from SARS-CoV-2) using DETECTR (Cas12a-based method exploiting *trans*-cleavage) and iCOLUMBO (Cas9-based method exploiting strand displacement). Note that the strip is used differently in both cases.

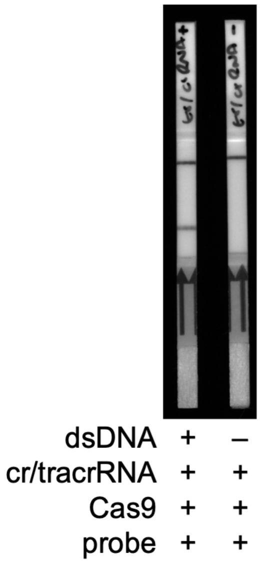

Fig. S4: Image of representative LFA strips in the detection of the E gene amplicon from SARS-CoV-2 using the bimolecule crRNA-tracrRNA. The use of this ribonucleoprotein does not degrade the ssDNA probe and the test line is colored properly in presence of the dsDNA input.

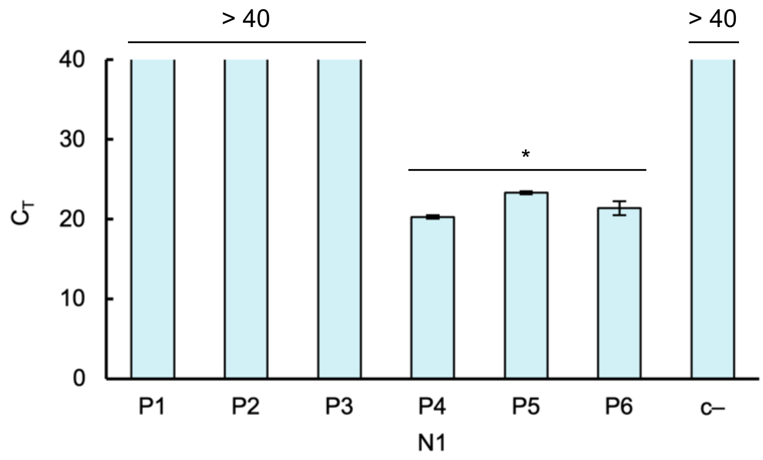

Fig. S5: RT-qPCR characterization of clinical samples [showing cycle threshold (C_T) values]. Samples with $C_T < 40$ (P4, P5, and P6) were considered positive for SARS-CoV-2 infection. Represented data correspond to means ± standard deviations (n = 3, technical replicates from a patient sample). *Statistically significant change (Welch's t-test, $P < 0.05$).

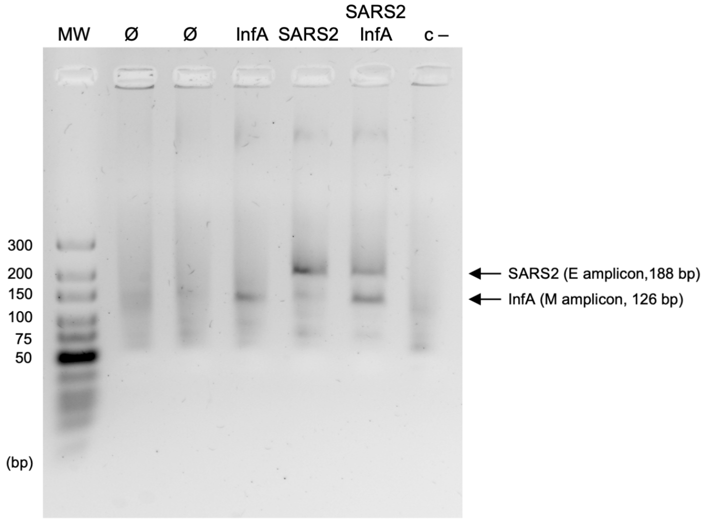

Fig. S6: Gel electrophoretic assay of the multiplexed RT-RPA of SARS-CoV-2 (E gene) and Influenza A/H1N1 (M gene) with spiked samples. Molecular marker, GeneRuler ultra-low range DNA ladder.

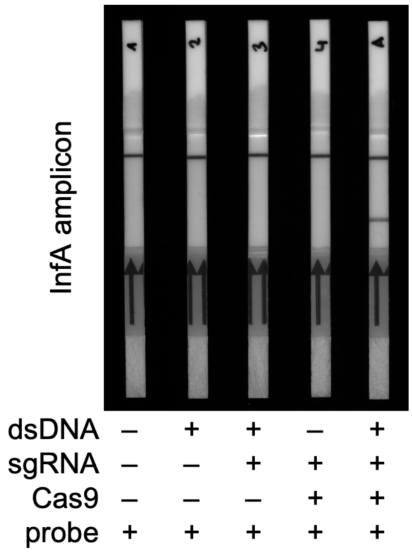

Fig. S7: Image of representative LFA strips in the single detection of Influenza A/H1N1 (RNA genome), coupling the pre-amplification step with Cas9-mediated strand displacement.

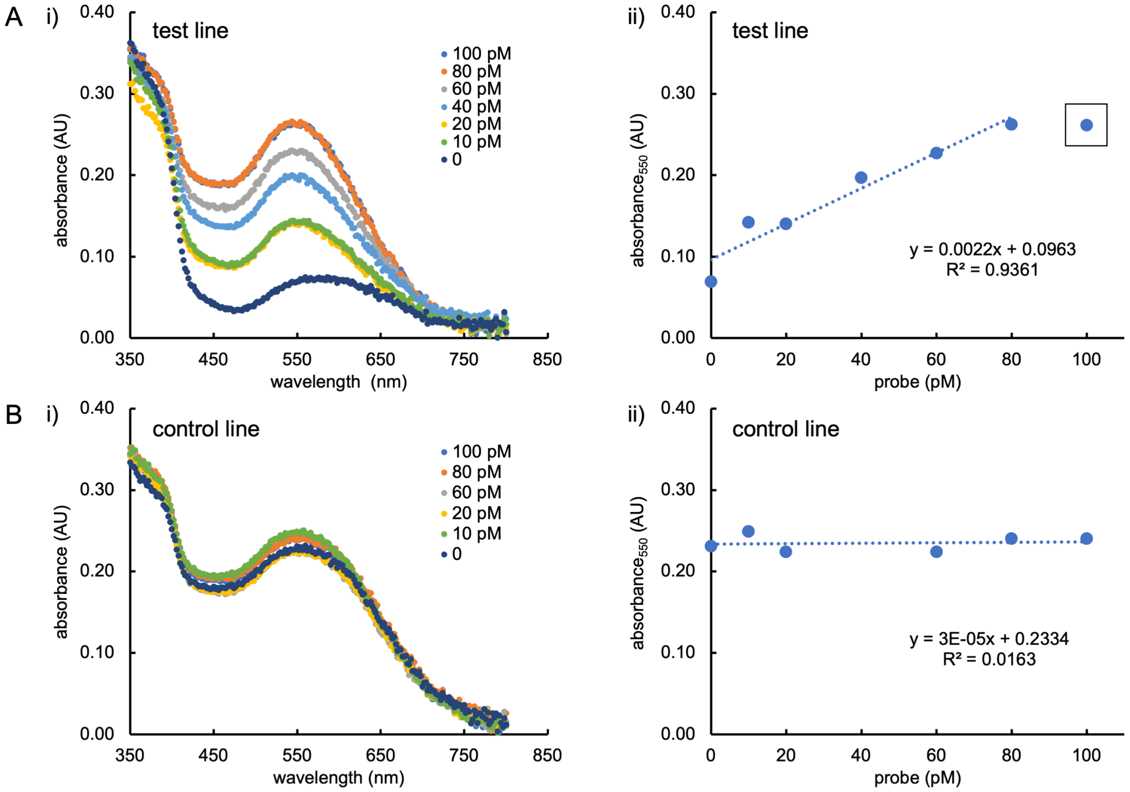

Fig. S8: Characterization of the sensitivity of the LFA strips in a low concentration regime[1]. A) Absorbance spectra of the test line (i) and linear regression between the absorbance at 550 nm and the probe concentration (ii). B) Absorbance spectra of the control line (i) and linear regression between the absorbance at 550 nm and the probe concentration (ii). Point within square was not considered for the regression.

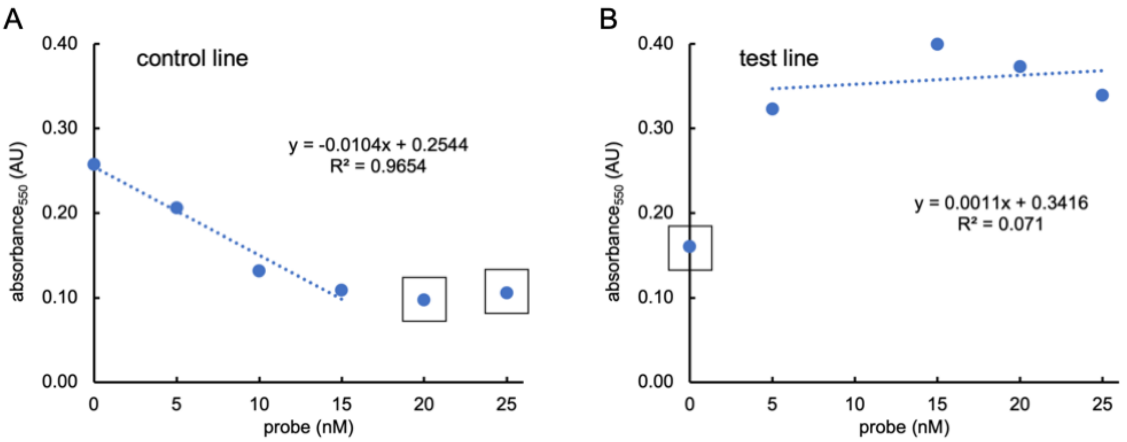

Fig. S9: Characterization of the sensitivity of the LFA strips in a high concentration regime[2]. A) Linear regression between the absorbance at 550 nm and the probe concentration in the control line. B) Linear regression between the absorbance at 550 nm and the probe concentration in the test line. Points within squares were not considered for the regressions.

---

[1]To evaluate the sensitivity of the test and control lines, we prepared different mixtures of fluorescein, biotin, and a dual-labelled probe (fluorescein and biotin), while fixing the total concentration of biotin at 100 pM (low concentration regime). For example, the first condition was 100 pM fluorescein, 100 pM biotin, and 0 pM probe. The second condition was 90 pM fluorescein, 90 pM biotin, and 10 pM probe. These preparations were run in triplicate on the LFA strips and the absorbance spectra were measured, revealing an absorbance peak at 550 nm due to the bound gold particles. Part of the strip where there is no line was used as blank for these measurements. The test line demonstrated superior sensitivity, allowing for the detection of subtle changes in concentration and becoming saturated at 80 pM.

[2]We also prepared different mixtures of fluorescein, biotin, and a dual-labelled probe (fluorescein and biotin), while fixing the total concentration of biotin at 25 pM (high concentration regime). For example, the first condition was 25 nM fluorescein, 25 nM biotin, and 0 pM probe. The second condition was 20 pM fluorescein, 20 pM biotin, and 5 pM probe. In this regime, the control line showed certain ability to discriminate concentrations.

Table S1: Sequences of all nucleic acids used in this work.

RT-RPA Oligos

Virus	Gene	Primer	Sequence 5' - 3'	5' modification
SARS-CoV-2	Nucleocapsid	N Forward	GGGTAGTCTTGTAGTGCGTTGTTCGTTCTATG	
SARS-CoV-2	Nucleocapsid	N Reverse	GTTCTCCATTCTGGTTACTGCCAGTTGAAT	B7
SARS-CoV-2	Envelope	E Forward	ACAGGTACGTTAATAGTTAATAGCGT	B7 or DIG
SARS-CoV-2	Envelope	E Reverse	GGAACTCTAGAAGAATTCAGATTTTTAACAC	
Influenza A/H1N1	Matrix 1	I Forward	CAGGCATGAAAACAGAATGGTGC	
Influenza A/H1N1	Matrix 1	I Reverse	GCATGTACCATCTGCCTAGTCTGATTAGC	B7

RT-qPCR Oligos

Virus	Gene	Primer	Sequence 5' - 3'	5'/3' modification
SARS-CoV-2	Nucleocapsid	N1 Forward	GACCCCAAAATCAGCGAAAT	
SARS-CoV-2	Nucleocapsid	N1 Reverse	TCTGGTTACTGCCAGTTGAATCTG	
SARS-CoV-2	Nucleocapsid	N1 probe	ACCCCGCATTACGTTTGGTGGACC	FAM/BHQ1

Guide RNAs

Virus	Gene	Cas protein	type	Sequence 5' - 3'
SARS-CoV-2	Nucleocapsid	Cas9	sgRNA	ggAAAAUUAGCGAAAUGCACCCCGCAUUACGUUGUUUUAGAGCUAGAAAUAGCAAGUUAAAAUAAGG CUAGUCCGUUAUCAACUUGAAAAAGUGGCACCGAGUCGGUGCUUUU
SARS-CoV-2	Envelope	Cas9	sgRNA	ggAGUAGUACGCACACAAUCGAAGCGCAGUAGUUUUAGAGCUAGAAAUAGCAAGUUAAAAUAAGGCU AGUCCGUUAUCAACUUGAAAAAGUGGCACCGAGUCGGUGCUUUU
SARS-CoV-2	Nucleocapsid	Cas12a	sgRNA	ggUAAUUUCUACUAAGUGUAGAUGUGGACCCUCAGAUUCAACU
Universal	-	Cas9	tracrRNA	ggUUCAAAACAGCAUAGCAAGUUAAAAUAAGGCUAGUCCGUUAUCAACUUGAAAAAGUGGCACCGAG UCGGUGCUUC
SARS-CoV-2	Envelope	Cas9	crRNA	ggAGUAGUACGCACACAAUCGAAGCGCAGUAGUUUUAGAGCUAUGCUGUU
Influenza A/H1N1	Matrix 1	Cas9	sgRNA	ggAACAGAUGGUUGGAUCGAGUGAACGUUUUAGAGCUAGAAAUAGCAAGUUAAAAUAAGGCUAGUCC GUUAUCAACUUGAAAAAGUGGCACCGAGUCGGUGCUUUU

CRISPR Probes

Virus	Gene	Cas protein	Sequence 5' - 3'	5' modification	3' modification
SARS-CoV-2	Nucleocapsid	Cas9	GCGAAATGCTGTAATGCGGGGTGCATTTCGC	FAM	
SARS-CoV-2	Envelope	Cas9	CGCACACAACTGCGCTTCGATTGTGTGCG	FAM	
Universal	-	Cas12a	TTATT	FAM	B7
Influenza A/H1N1	Matrix 1	Cas9	GATGGTTCGATCCAGCCATC	FAM	
